# MolPrice: assessing synthetic accessibility of molecules based on market value

**DOI:** 10.1186/s13321-025-01076-3

**Published:** 2025-09-29

**Authors:** Friedrich Hastedt, Klaus Hellgardt, Sophia Yaliraki, Dongda Zhang, Antonio del Rio Chanona

**Affiliations:** 1 Department of Chemical Engineering, Imperial College London, London, UK; 2 Department of Chemistry, Imperial College London, London, UK; 3 Department of Chemical Engineering, University of Manchester, Manchester, UK

**Keywords:** Synthetic accessibility, Molecular price, Virtual screening, Self-supervised learning

## Abstract

Machine learning approaches for conceptualizing and designing in silico compounds have attracted significant attention. However, the applicability of these compounds is often challenged by synthetic viability and cost-effectiveness. Researchers introduced proxy-scores, known as synthethic accessiblity scoring, to quantify the ease of synthesis for virtual molecules. Despite their utility, existing synthetic accessibility tools have notable limitations: they overlook compound purchasability, lack physical interpretability, and often rely on imperfect computer-aided synthesis planning algorithms. We introduce *MolPrice*, an accurate and fast model for molecular price prediction. Utilizing self-supervised contrastive learning, *MolPrice* autonomously generates price labels for synthetically complex molecules, enabling the model to generalize to molecules beyond the training distribution. Our results show that *MolPrice* reliably assigns higher prices to synthetically complex molecules than to readily purchasable ones, effectively distinguishing different levels of synthetic accessibility. Furthermore, *MolPrice* achieves competitive performance on literature benchmarks for synthetic accessibility. To demonstrate its practical utility, we conduct a virtual screening case study, illustrating how *MolPrice* successfully identifies purchasable molecules from a large candidate library. *MolPrice* bridges the gap between generative molecular design and real-world feasibility by integrating cost-awareness into synthetic accessibility assessment, making it a powerful model to accelerate molecular discovery.

**Supplementary Information:**

The online version contains supplementary material available at 10.1186/s13321-025-01076-3.

## Scientific contribution

We introduce* MolPrice*, a machine learning model that predicts molecular price as a proxy for synthetic accessibility. Unlike existing approaches,* MolPrice* integrates cost-awareness into accessibility assessment, allowing it to distinguish readily purchasable molecules from synthetically complex ones. The model is computationally efficient, and our results demonstrate its suitability for large-scale virtual screening. This work thus provides a practical tool to prioritize cheap and synthetically viable compounds in early stage discovery workflows.

**Supplementary Information:**

The online version contains supplementary material available at 10.1186/s13321-025-01076-3.

## Introduction

Generative modeling of molecules represents one of the most promising applications of Machine Learning (ML) in chemistry [1]. By leveraging advanced computational techniques, these models can rapidly propose novel drug molecules with targeted physical properties in mere seconds [2, 3]. Despite their potential, a critical challenge persists: how to ensure the practical synthesizability of these computationally generated molecules [4]. With the emergence of Computer-Aided Synthesis Planning (CASP) [5], scientists can perform retrosynthesis on the virtual molecule in an automated manner. This allows for the discovery of synthesis pathways and facilitates wet-lab synthesis of the virtual molecule. While CASP is a powerful tool, it is “slow” at inference time, taking about 1-3 min to predict synthesis pathways for a single molecule. In generative design routines (and also virtual screening), one often screens thousands or millions of different molecules. For example, to evaluate the synthesizability of 1 million molecules (which is comparable in size to the ChEMBL database [6] of bioactive molecules), CASP would take 2 years to complete the task (assuming non-parallelization and 1 min per molecule). It is clear that CASP does not scale to these dimensions [7]. Even if CASP leads to the successful identification of synthesis routes, one still has to identify the economic viability of these paths in terms of scale-up potential and sustainability [8]. Although CASP has yielded successful results [9], the procedure remains time-consuming and often requires additional thermodynamic insights that extend beyond the direct output of CASP.

Instead of subjecting all molecules to CASP, researchers proposed proxy scores capturing the synthesizability of a molecule. Herein, synthesizability refers to the likelihood of successful experimental synthesis for a given molecule. These scoring indices assess the Synthetic Accessibility (SA) of a molecule within milliseconds. Often treated as a binary classification problem [10, 11], the SA tools classify molecules as *Easy-to-Synthesize* (ES) or *Hard-to-Synthesize* (HS). Molecules that fall within the latter category are discarded. ES molecules can then be subjected to further CASP.

### Previous work

Existing SA methods are either (i) stucture-based [11, 12] or (ii) retrosynthetic/reaction-based [7, 10, 13–16].

Structure-based methods estimate the ease of synthesis of a molecule based on its complexity indicators [17] and functional groups. For example, *SAScore* [12] assigns a score depending on the presence/absence of specific functional groups, macrocycles, stereocentres and the overall size of the molecule. Structure-based indices have been used as an objective for generative design [18] and as a post-hoc evaluation metric for fragment-based discovery [19]. It is to note that complexity indicators [20] may correlate poorly with synthetic feasibility as is often the case for natural products. Depending on the chemical space of interest, the assumption that more complex molecules are less synthetically accessible is unlikely to hold [21].

Retrosynthetic-based approaches aim to predict specific outputs of a (chosen) CASP tool. For example, Kim et al. [15] proposed *DRFScore* to predict the number of reaction steps within a synthesis route. Other approaches focus on developing SA indices that predict the likelihood of successful synthesis planning [10, 16, 21]. In these cases, success is defined by whether CASP can find at least one synthesis pathway. This effectively transforms the problem into binary classification: a molecule is either easily synthesizable if the CASP tool finds a synthesis route, or hard-to-synthesize if it does not.

It is important to note that these SA indices employ different criteria to define HS molecules. In Refs. [10, 16, 21], molecules are classified as HS when CASP fails to find a route within a predetermined computational budget. In contrast, *DRFScore* considers molecules as HS if they require more than a maximum number of reaction steps N_max_.

Both methodologies have their own merits, but face similar limitations: The calculated SA scores are neither interpretable nor reflective of the actual price of the molecule [22]. This is because the score is often binary in nature or limited to a user-defined scale (e.g., 1-5 for *SCScore* [23] or 1-10 for *SAScore* [12]). Furthermore, the ES database is obtained from databanks such as ZINC20 [24] or ChEMBL [6]. While molecules in these datasets are most likely easy-to-synthesize, they may not always be readily available or their prices may be expensive. Similarly, molecules in the HS database may appear synthetically inaccessible, but are purchasable from a proprietary supplier (and could exists as a natural product). Lastly, retrosynthetic-based tools depend on the accuracy of the underlying CASP, whose accuracy in return is dependent on its training dataset and its hyperparameters as well as the specific single and multi-step models used for the planning procedure [25, 26]. Thus, these shortcomings and flaws are inherent in the SA tool.

This work is inspired by Sanchez-Garcia et al. [22], who proposed to evaluate the synthetic accessibility of a molecule using its price as a proxy. The idea is intuitive: A higher price implies a higher cost of synthesis (e.g., expensive reagents or high energy usage), and vice versa for a lower price. Their model for price prediction, *CoPriNet*, takes the 2D molecular graph as an input and predicts the logarithm of price in USD/mmol as an output. For training, they used Mcule’s [27] database consisting of 6 M molecules along with their market price. However, *CoPriNet* experiences a significant limitation: It struggles to generalize to out-of-distribution HS molecules. This comes from the fact that all molecules in the training dataset are easy-to-synthesize (the molecules are purchasable) and the model does not “see” any HS molecule during training. As a result, the model assigns a similar price value to ES and HS molecules. For this reason, the authors state that *CoPriNet* cannot be used as a standalone SA scoring tool. Instead, it should be used in conjunction with other indices such as *SAScore* [22].

### Contributions

To overcome existing shortcomings of SA scoring techniques and enable an interpretable SA index based on economic viability, we introduce *MolPrice*. *MolPrice* is an accurate and fast ML model for molecular price prediction trained on a database of 5.5M molecules extracted from Molport [28], an advanced chemical market place for purchasing chemicals from reliable suppliers. Using a contrastive learning approach, *MolPrice* self-generates price labels for unlabeled HS molecular structures. Particularly, it learns to distinguish HS molecules from those found in the purchasable (ES) dataset. Our comprehensive investigation of various molecular representation techniques reveals a significant insight: substructural features (such as functional groups) exhibit a strong correlation with market prices. This finding supports the notion that synthetic complexity directly translates into economic value.

Our case study demonstrates that *MolPrice* robustly predicts molecular prices while providing reliable synthetic accessibility estimates, making it valuable for both virtual screening and de novo molecular design.

## Methodology

### Data extraction and preprocessing

Molport [28] provided the modeling data as a snapshot of their purchasable database in March 2024. The database stores approximately 5.5M molecules that are readily available from a range of suppliers. For each molecule in the database, a price in USD per unit weight is provided for a variety of suppliers. To arrive at the training dataset, the following steps were followed:

1. Discard molecules that cannot be read by RDKit [29]
2. Normalize all molecular prices to USD per mmol, filtering molecules with missing weight units. The unit is chosen in molar basis (instead of mass), as it was found to have stronger correlation with SA measurements [22]
3. Obtain the molecule with the minimum price in USD per mmol
4. Convert prices to (natural) logarithmic scale. Prices range from [0,12] - distribution shown in Figure S1 (in the SI)
5. Remove molecules with price < 2 USD per mmol. These molecules are either stabilizing salts, metals or solvents

These steps filter out $\sim$30000 molecules, which translates to 0.5% of the entire dataset. Over 90% of molecules are filtered out in step 5 of the procedure. Furthermore, molecules that were discarded by RDKit in step 1 were found to be chemically invalid. The dataset is randomly split into (0.9,0.05,0.05) subsets for training, validation and testing, respectively. A higher training split was chosen because of the large abundance of data leaving over 500k molecules for testing and validation alone. A single split was deemed sufficient according to the information provided in recent studies on significant method comparison protocols for property prediction [30].

### Model development

#### Molecular representation

**Fig. 1:**
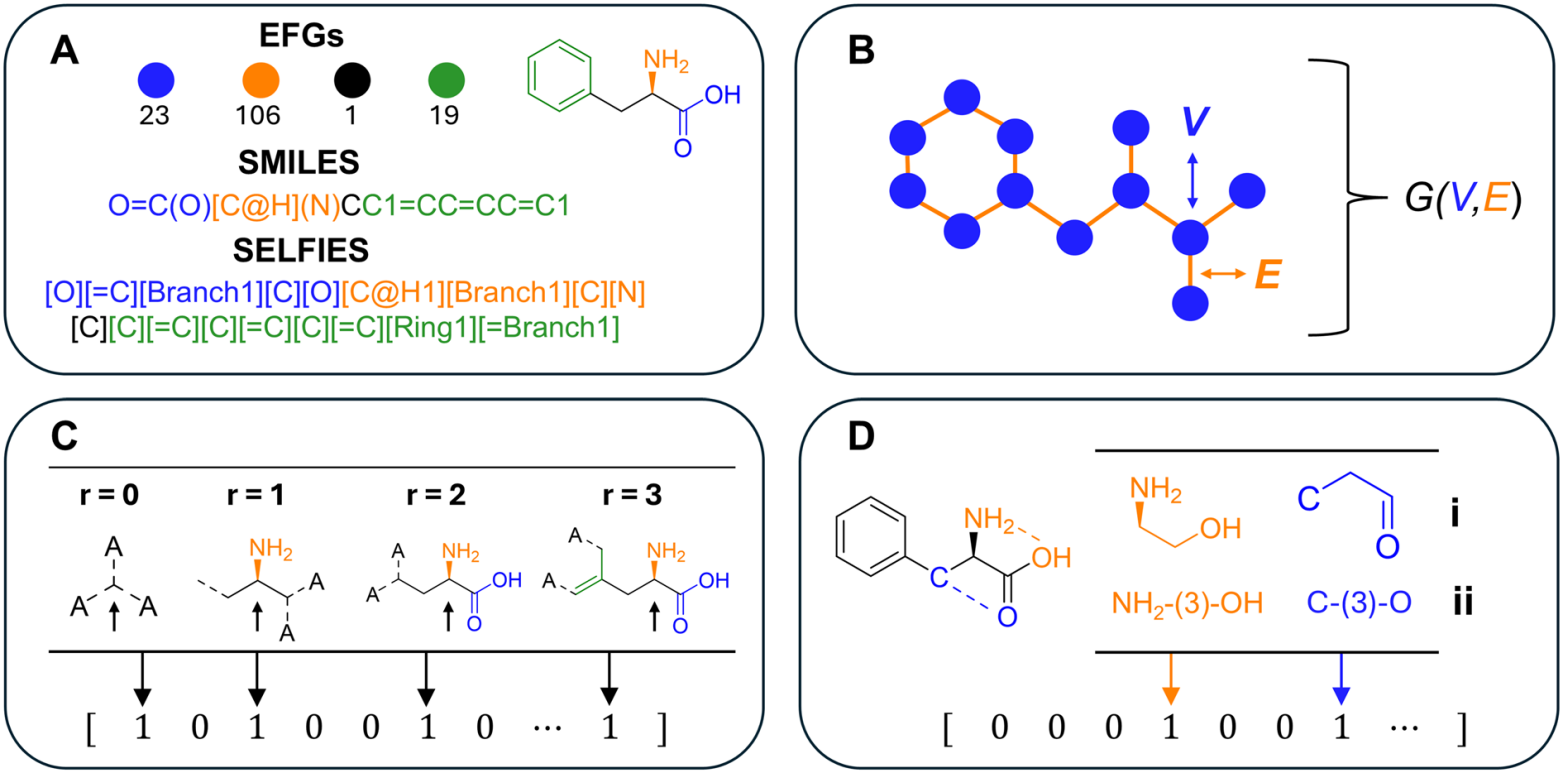
An overview of different molecular representation. **A** Extended Functional Groups (EFGs) split the molecule into substructures according to predefined rules. A unique integer number is assigned to each substructure. SMILES and SELFIES are text-based representations of a molecule. **B** 2D molecular graph with node features (*V*) and edge features (*E*). The graph is numerically represented by its connectivity alongside *G(V,E)*. **C** Circular fingerprint encodes substructures in the molecules up to user-defined radius *r*. The example focuses on the amine-adjacent carbon up to radius $r=3$. These substructures are hashed to a binary vector. **D** **i.** Path-based and **ii.** Atom-pair fingerprints encode sequences or pairs of atoms, respectively. Two examples are shown, the first encoding the pair of the amine and hydroxyl groups with 3 bonds between them. Similar to the circular fingerprint, atoms are hashed to a binary vector. The graphic is inspired by Ref. [31]

Property prediction models can be based on a variety of molecular features and machine learning architectures, the most effective depending on the specific downstream application. We explore different featurization schemes in this work as illustrated in Fig. 1.

This allows us to not only discover relationships between specific molecular features and economic value, but also find the most prominent model. We aim to answer whether features such as functional groups or larger molecular moieties influence the molecular price. For the interested reader, an outline of different featurization methods is provided below.

Model hyperparameters such as hidden dimensions and learning rates are provided in the SI.

- **Text-Based Representation** such as SELFIES [32] and SMILES [33] encode the molecule using tokens (letter-like symbols). Inspired by advances in Natural Language Processing (NLP), SMILES and SELFIES can be used as an input for ML algorithms. We utilize SMILES as an input to the vanilla Transformer and a pre-trained RoBERTa model (ChemBERTa) by DeepChem [34], which is a widely utilized model for molecular property prediction.
- **Molecular Graphs** are a 2D or 3D representation of the molecule. Each atom and bond in the graph is described as a node and edge, respectively. Node and edge features are user-selected, often based on prior experience or intuition. We use the literature price prediction model, *CoPriNet* [22], as a baseline GNN (Graph Neural Network). *CoPriNet* uses nodes features such as atomic number, valence and aromaticity and edge features such as bond type, conjugation and ring membership. From these features, the model can learn relevant information such as presence of aromatic substructures and functional groups.
- **Molecular Fingerprints** encode the structure of the molecule in a bit vector of user-defined length. Each “on” bit in the vector highlights the presence of a particular substructure in the molecule. Various fingerprint encoding methods exist, e.g., based on circular or path-based substructures and pairs of atoms. We select four different fingerprint generators for this study: (1) Morgan (ECFP) and (2) SECFP (derived from the MinHash fingerprint constructor [35]) are circular fingerprints that encode molecular substructures up to a fixed radius *r*. (3) Atom-pair encodes pairs of atoms along with the shortest distance between them. (4) Path-based (RDKit) encodes short-range information of a sequence of atoms found within the same path. In short, the circular fingerprints are better at encoding functional groups, while atom-pair and path-based fingerprints capture backbone patterns more efficiently. The different fingerprints are used as input features to a multilayer perceptron (MLP).
- **Functional Groups** algorithms detect molecular functionalizations based on pre-defined rules. Ertl [36]’s algorithm detects functional groups based on the connectivity of heteroatoms and bond types. An extension to Erlt’s algorithm was proposed in Ref. [37], known as extended functional groups (EFGs). We use the EFG algorithm to construct a vocabulary of functional groups in our database. The algorithm detected $>17,000$ distinct functional groups, highlighting the diversity within the dataset. For each molecule, the EFG algorithm then detects the molecule’s functional groups and looks up the vocabulary to assign integer values (according to the position of the groups in the vocabulary). Thus, each molecule has a sequence of integers representing its functional groups. To construct a numerical representation from this sequence, we use a long-short term memory (LSTM) network (and a learnable embedding matrix). This was preferred over Transformer models, due to its faster training and inference speed. As a note, the functional group encoding could be used as a molecular fingerprint vector. We decided against it since the resulting bit vector fingerprint is extremely sparse, that is, for most molecules, $<10$ bits are “on” with an overall fingerprint dimensions of $>3000$ translating to $<0.3\%$. It may be worth investigating sparse machine learning techniques instead of the LSTM in the future.

The models are trained on the Mean-Squared Error (MSE) loss. Once the best model is identified, its hyperparameters (HP) were tuned using Optuna’s Tree-structure Parzan Estimator [38]. The Parzan Estimator fits a surrogate model (to be more precise, a Gaussian mixture) and finds the best set of hyperparameters using Bayesian optimization. The result of the HP tuning is presented in the SI. All models were built with PyTorch and PyTorch Lightning [39].

#### Evaluation metrics

For the regression tasks, we use the MSE, coefficient of determination ($\hbox {R}^\text {2}$) and Spearman’s correlation coefficient ($\hbox {r}_\text {s}$) as evaluation metrics. The Spearman coefficient evaluates the model’s ability to correctly rank the order of molecules from cheap to expensive. Additionally, inference time is reported for the different algorithms. SA routines generally require millions of calls to the SA oracle. A small difference in inference time (e.g., 1 ms/mol vs 5 ms/mol) can therefore lead to a significant difference in the exploration time.

For binary SA classification tasks, we employ Matthew’s correlation coefficient (MCC), area under receiver operating characteristic curve (ROC-AUC) and the F-score. All metrics provide a measure for the model’s ability to distinguish between ES and HS molecules. The F-score and MCC focus on the classification performance for an optimized threshold, while ROC-AUC measures the ability to distinguish between classes at all thresholds. The threshold is a certain price below which the molecules are classified as ES (and vice versa for HS). All metrics are commonly used for binary classification problems.

### Self-supervised learning: differentiating synthetic complexities

Learning the difference between synthetically complex (HS) and purchasable (ES) molecules is key to steer the price prediction model towards SA measurement. After training on the purchasable database, the model will have only seen ES molecules. Thus, it is unable to generalize to synthetically complex molecules outside its training distribution. A higher price assigned by the pre-trained model would therefore indicate synthetic inaccessibility in current market conditions, rather than synthetic difficulty (in terms of reactivity).

**Fig. 2:**
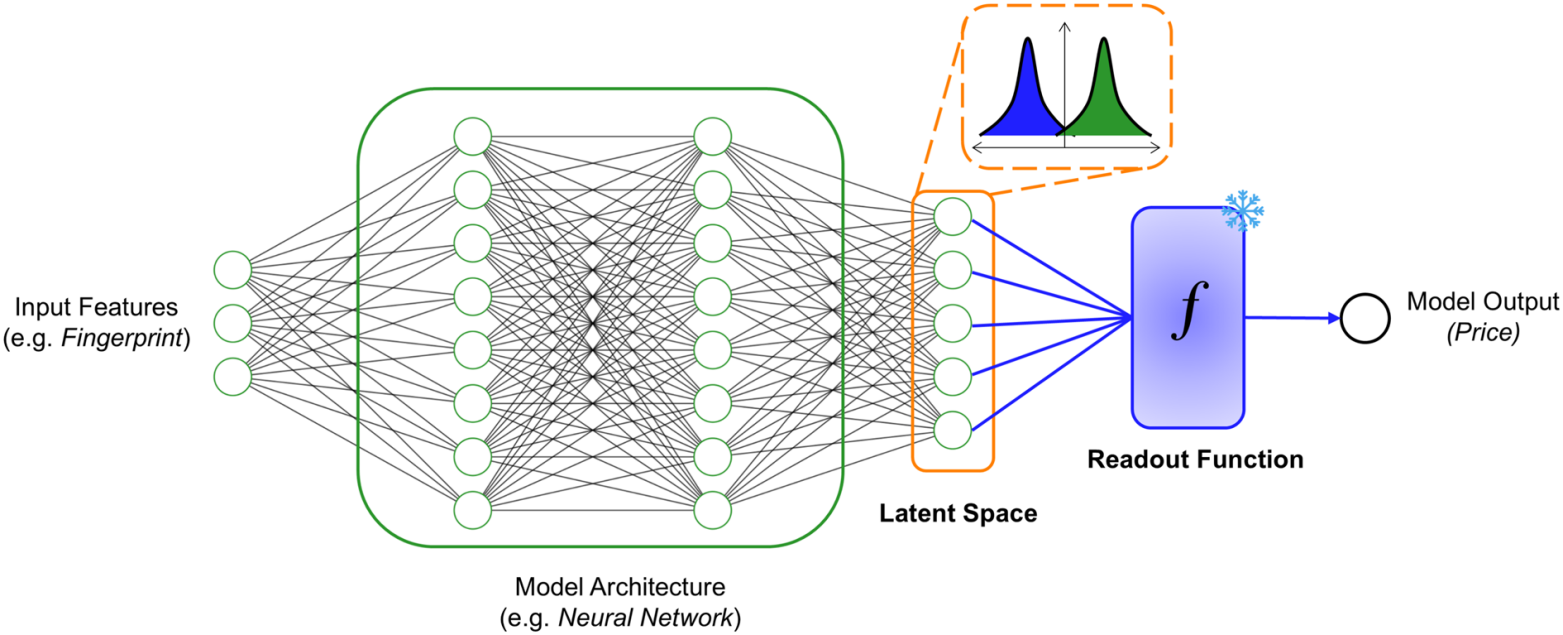
Visualization of contrastive learning approach. The input features are transformed into a numerical latent space by the model architecture. The contrastive loss separates the two molecular classes in the latent space by minimizing the overlap of their distributions. The readout function computes the molecular price from its latent space

Obtaining price labels for HS molecules is challenging because they are not offered by suppliers or have not even been synthesized yet. The model has to learn HS labels by itself. To do so, we introduce a contrastive learning approach based on the latent space of the model. Herein, the latent space refers to the numerical representation of the molecule prior to the final readout function *f*. The readout function is typically a small neural network or linear weight matrix to transform the numerical (latent) representation into the model output as $f:\mathbb {R}^N \rightarrow \mathbb {R}$, where *N* is the latent dimension. A visual representation is shown in Fig. 2.

Our contrastive learning approach works on two principles: First, it requires a trained model on the respective model output (molecular price). Given an accurate (pre-trained) model, the weights in the readout function *f* are frozen. This is because, we assume that the model has found an accurate relationship between the numerical representation of the molecule (latent space) and the its price, with the information being stored in the readout function. This means that only the parameters in the model architecture need to be updated to learn a suitable latent representation for the HS molecules. It is important to note that although the model weights are fine-tuned during contrastive learning, the latent representations of ES molecules remain close to their original (pre-trained) values due to the influence of the MSE loss (Eq. 1), which penalizes deviations in price predictions. As a result, the mapping from latent space to price via the frozen readout function remains accurate for ES molecules. Additionally, minor shifts in individual latent dimensions are balanced by adjustments in other related dimensions (see SI Figure S2).

Second, we assume that each dimension in the latent space ($j \in N)$ follows a univariate Gaussian distribution. The second assumption allows us to construct the semi-supervised contrastive loss as:

1 $$\begin{aligned} L&= \frac{1}{M}\sum _{i \in ES}(y_i-\hat{y}_i)^2 +\lambda \cdot \frac{1}{N}\sum _{j=0}^N H_j \; ,\end{aligned}$$

2 $$\begin{aligned} H_j&= \sqrt{\frac{2\sigma _{ES,j}\sigma _{HS,j}}{\sigma _{ES,j}^2+\sigma _{HS,j}^2}}\exp \left( -\frac{1}{4}\frac{\left( \mu _{ES,j}-\mu _{HS,j}\right) ^2}{\sigma _{ES,j}^2+\sigma _{HS,j}^2}\right) \; , \end{aligned}$$

where *M* and *N* are the dataset size and latent dimensions, respectively. $H_j$ is the analytical Hellinger affinity (or Bhattacharyya coefficient) between two univariate Gaussian distributions and is computed individually for all latent dimensions ($\forall j \in N$). The Hellinger affinity measures the degree of separation between two Gaussian distributions $\mathcal {N}(\mu _{ES},\sigma _{ES}^2)$ and $\mathcal {N}(\mu _{HS},\sigma _{HS}^2)$ for ES and HS molecules, respectively. The affinity tends to a minimum value of 0 when there is a maximum dissimilarity between the ES and HS latent space representation. On the other hand, if the distributions are the same, it tends to a maximum value of 1. Thus, minimizing the Hellinger affinity naturally leads to a different numerical latent representation of ES and HS molecules. Given the frozen weights in the readout function, a different latent representation should translate to a different price label for ES and HS molecules. The MSE loss in Eq. 1 acts as a regularization term that anchors the latent representations of ES molecules, preserving the learned mapping of the frozen readout function. We introduce a hyperparameter $\lambda$ that controls the loss magnitude between the MSE and Hellinger loss. We set $\lambda$ to 0.05 by default, which was found to work well through experimentation. Increasing $\lambda$ to larger values can lead to either a decremental performance on the MSE loss (if the MSE and Hellinger losses are conflicting objectives) or to an overly fast convergence to a local optima.

Finally, a soft constraint can be introduced in Eq. 1 to add a physical upper bound on the HS price labels as $\frac{1}{L}\sum _{i\in HS}\max (0, y_i-X)$, where $y_i$ is the predicted price of the molecule and *L* is the size of the HS dataset. This soft constraint adds a penalty term to the loss function in case any molecular price exceeds a value of *X*. We initially set the default upper bound to $X=20$, an unrealistically high value, which incorporates the idea that molecular prices saturate and do not increase monotonically to infinity. However, we found that the constraint was not necessary for model convergence (predicted prices for HS range from 10 to 15). If one has any physical information about an accurate upper bound, one could activate this constraint. Similarly, one could set a lower bound for HS prices. We did not investigate this further since it was of interest to see whether the model itself would price HS molecules higher than the ES counterpart.

As a note, there is no theoretical guarantee that the learned price labels for the HS molecules exceed the ES labels. Our contrastive loss function is solely based on the latent space and therefore, the model could equivalently learn to price HS molecules lower than ES molecules. We hypothesize that this is however unlikely, as the model is expected pick up on the overall trend from the ES labeled dataset: a more synthetically complex molecule is likely to be priced higher on the market.

The dataset of HS molecules is obtained from Ref. [10] including 400,000 molecules. The molecules in this dataset were generated by the Nonpher algorithm [40] and are known to be HS. However, our methodology is not linked to a specific dataset. Taking the natural products from the COCONUT database [41], we demonstrate that *MolPrice* can also learn to distinguish between purchasable (ES) molecules and natural products (see SI). In this case, the model is not learning molecular differences in terms of synthetic complexity, but rather distinguishing between the structural features of purchasable molecules and those of complex natural products [42].

### Model testing

For the price prediction task, the model is tested on the hold-out test set consisting of 250k molecules. To emphasize the use of *MolPrice* as an SA tool, we compare *MolPrice* to state-of-the-art SA tools [10–12, 14, 16, 23]. In particular, we take test sets 1-3 (TS1-3) from Ref. [10]. TS1 contains $\sim$3500 ES and HS compounds from the ZINC15 [24] and GDB-17 [43] databases. TS2 contains $\sim$30000 molecules from the ChEMBL [6] and GDB databases and labels them as either ES or HS. TS3 consists of 900 ES and 900 HS molecules, collected from a variety of sources [10]. It is known to be the most challenging of the 3 datasets since the compounds in the ES/HS datasets are more similar. The binary labeling of molecules in TS2/3 is achieved via retrosynthesis software (Retro*) [44].

Furthermore, we investigate whether the addition of complexity indicators computed via RDKit [29] improves *MolPrice*’s performance for SA prediction. This is because molecular fingerprints are good at capturing 2D information but fail at encoding 3D factors such as presence of stereocentres [17]. The resulting hybrid fingerprint is constructed by concatenating the complexity indicators to the molecular fingerprint (refer to SI for details on complexity indicators).

#### Virtual screening study

We verify the effectiveness of *MolPrice* on a multi-objective virtual screening case study using *MolPAL* [45]; an active learning algorithm for exploring molecular virtual libraries. Particularly, the objective is to find ligands (from a virtual library) that are dual inhibitors of proteins IGF1R and EFGR (a “potential starting point for esophageal cancer” [45]). The molecular price calculated by *MolPrice* provides a third objective as a proxy for SA measurement. Thus, *MolPAL* aims to return ligands that not only exhibit good profiles for the two proteins but also are purchasable.

As docking scores are expensive to compute, *MolPAL* only acquires a fraction of the virtual library as candidate ligands. This is done through a multi-objective Bayesian optimization routine (the reader is referred to Ref. [45] for detailed information). We compare the candidate ligands returned by *MolPAL* with and without using price as a proxy for SA. We refer to the two results as SA-aware (3 objectives) and -unaware (2 objectives), respectively. The candidate ligands are selected from a virtual library consisting of 260k molecules from the DOCKSTRING benchmark [46].

For the SA-aware case, we expect *MolPAL* to return candidate ligands that are synthetically accessible. To verify that this is indeed the case, we calculate quantitative statistics for both the SA-aware and -unaware cases in two ways: First, we query the Mcule API [27] (website for finding chemical suppliers) to compare the selected candidate ligands to readily available molecules. Second, we compute synthesis routes for all candidate ligands using ASKCOS v2 [47] (settings for ASKCOS are provided in the SI). From the synthesis routes, we can calculate informative metrics such as average route length and confidence scores.

## Results & discussion

### Molecular price prediction

Table 1 presents the test set results for molecular price prediction (prior to the self-supervised learning). The aim is to obtain an accurate model for the regression task. Among the various models, the neural network (MLP) using Morgan / SECFP fingerprints achieves the best performance in MSE, R^2^, and Spearman’s rank correlation (r_s_) on the test set. This suggests that Morgan/SECFP fingerprints offer slightly superior molecular representation compared to Atom Pair and Path-based fingerprints for the price prediction task. The likely reason for this advantage lies in their construction method — both Morgan and SECFP fingerprints encode molecular structures using circular atom-based environments, effectively capturing functional group (substructure) information. In contrast, Atom Pair and Path-based (RDKit) fingerprints emphasize atomic sequences, making them less effective at encoding fine-grained molecular substructures for the task at hand. The literature GNN model, *CoPriNet* [22], demonstrates performance close to that of the MLP models using circular fingerprints. This similarity may arise from the underlying message-passing mechanism in GNNs, which resembles the circular encoding strategy of fingerprint generation. Each atom in the molecule obtains information from its neighbouring atoms in a single message-passing operation. Stacking up *L* message-passing operations, an atom receives information from neighboring atoms that are *L* bonds away. This is comparable to the encoding radius *r* used by the circular fingerprints. As a result, both approaches leverage comparable structural information of the molecule.

The pre-trained Transformer model (RoBERTa) outperforms its vanilla counterpart, benefiting from prior exposure to SMILES-based molecular representations. However, neither SMILES-based model surpasses the fingerprint-based MLP nor the GNN. This limitation might arise because SMILES tokenization operates at the atomic level, lacking explicit molecular substructure information — a key feature captured by the MLP and GNN models. The extended functional group (EFG) LSTM model performs the worst out of all featurization schemes. Although EFGs aim to directly encode functional group information, they perform “poorly” attributed to its sparse high-dimensional feature space (as outlined in Methodology). Thus, it is equally important to select relevant features for a given task and to identify a suitable way to represent them numerically.

**Table 1:** Results for different molecular featurization on the hold-out test set. Performance metrics include regression accuracy (Mean Squared Error - MSE, coefficient of determination - R^2^, Spearman’s correlation coefficent - r_s_) and inference/training time

Architecture	Feature (Model)	MSE ($\downarrow$)	R^2^ ($\uparrow$)	r_s_ ($\uparrow$)	Inf. Time (ms/mol) ($\downarrow$)	Training time ($\downarrow$)
MLP	Atom pair	0.58	0.72	0.84	**1.3**	<**30 min**
	Path-based (RDKit)	0.61	0.71	0.84	**1.3**	<**30 min**
	Morgan	0.51	**0.76**	0.87	**1.3**	<**30 min**
	SECFP	**0.50**	**0.76**	** 0.88**	**1.3**	<**30 min**
Transformer	SMILES (Vanilla)	0.88	0.58	0.72	41.1	$\sim$ 1 day
	SMILES (RoBERTa)	0.58	0.72	0.85	29.1	$\sim$ 1 day
LSTM	EFGs	0.62	0.70	0.82	3.6	$\sim$ 2-3 hr
GNN	2D Graph (CoPriNet)	0.52	0.74	0.85	21.5	$\sim$ 1 day

The best value is highlighted in bold

The inference and training time emphasize the benefits of the fingerprint models. From Table 1, the MLP model stands out with an inference speed of 1.3 ms per molecule (evaluated on an Intel vPro i9 laptop CPU) and an approximate training time of less than 30 min (evaluated on NVIDIA RTX 6000). If the underlying ES database was updated, one could retrain the model in a reasonable time frame. In contrast, the inference time for *CoPriNet* and Transformer models is 20–40 times larger. The training itself demands several days, rendering retraining computationally expensive.

This analysis highlights that molecular representations incorporating substructural features - such as circular fingerprints (Morgan, SECFP) and GNN-based features — correlate well with market price. This finding reinforces the link between structural complexity and market valuation.

### Learning synthetic accessibility

**Fig. 3:**
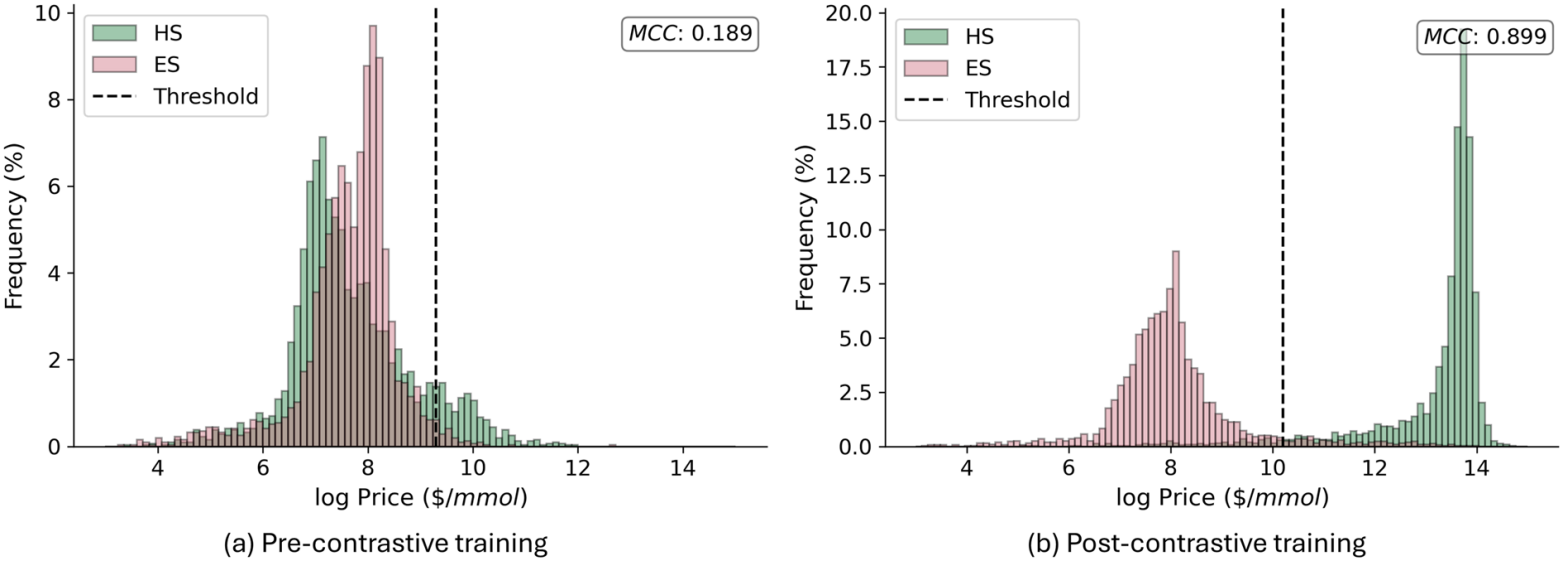
Predicted price for ES and HS molecules prior and post self-supervised (contrastive) learning using the SECFP Hybrid fingerprint

We take the best performing model from the price prediction stage (circular fingerprints) and use the trained model to continue training on the modified contrastive learning loss function. Following contrastive learning, the model is expected to distinguish ES and HS molecules based on its price label. Figure 3 shows the predicted price for ES and HS molecules before and post self-supervised learning. By visual inspection, it is clear that the model effectively learns to distinguish between ES and HS molecules according to their prices. In Fig. 3a, the pre-trained model assigns a similar price range to both ES and HS molecules. While the model could be used to assign a price to a new (synthesizable) molecule, it is evident that it cannot be used for SA measurement. In Fig. 3b, we observe that the model learns to assign higher prices to synthetically complex molecules by itself. This finding is based on empirical analysis and has no theoretical guarantee. Nonetheless, we trained a total of 4 different fingerprint models listed in Table 2 and recovered the same output for each experiment.

It is worth noting that although *MolPrice* is trained on synthetically complex molecules, it differs from reaction-based synthetic accessibility models like *RAScore* [21] or *BR-SAScore* [14], which learn directly from retrosynthetic analysis.

Table 2 provides a statistical comparison for the binary classification performance of the different circular fingerprints. In particular, we investigate both Morgan and SECFP fingerprints alongside their “hybrid” version, where (RDKit-generated) 3D molecular complexity indicators are added directly to the fingerprint (i.e., appended to the end of the fingerprint vector). All fingerprint models show promising results for the binary classification of ES/HS molecules. The SECFP fingerprint outperforms the Morgan fingerprint in terms of MCC, ROC-AUC and F-Score (the three binary classification evaluation metrics). It is also worth noting that the addition of complexity indicators improves the performance for both Morgan and SECFP fingerprints. The complexity indicators add global molecular information not encoded in the fingerprints, thus providing additional insights.

**Table 2:** Binary Classification Performance of different fingerprints on ES and HS datasets from Ref. [10]

Fingerprint	MCC ($\uparrow$)	ROC-AUC ($\uparrow$)	F-Score ($\uparrow$)	R^2^ ($\uparrow$)	Threshold
Morgan	0.841	0.966	0.920	0.736	9.87
Morgan Hyb.	0.875	0.977	0.938	0.732	10.86
SECFP	0.877	0.974	0.938	0.742	10.02
SECFP Hyb.	**0.899**	**0.982**	**0.950**	**0.746**	10.19

The price value at which the best binary classification is achieved is denoted as Threshold

The best value is highlighted in bold, second best value is underlined

All models retain their good performance on the initial price prediction task, underpinned by the high R^2^ values. A small drop is expected as the two loss terms in Eq. 1 will be conflicting for some molecules in the dataset. The Morgan Hybrid fingerprint experiences the “largest” decline in predictive performance from 0.76 to 0.73. This is because both the MSE and contrastive loss objectives were found to be opposing and in return, we increased $\lambda \rightarrow 0.3$. A carefully chosen $\lambda$ value thus ensures a good performance on both the classification task, while retaining an accurate model for price prediction.

#### Latent space analysis

We analyze the latent space of the model to corroborate the effective separation of the ES/HS molecules. The latent space consists of 10 dimensions, which is the same for all models investigated. While this can be considered a small latent space (in the realm of ML), it also increases the likelihood that each dimension carries distinct information and decreases the likelihood of redundant dimensions (known as superposition).

**Fig. 4:**
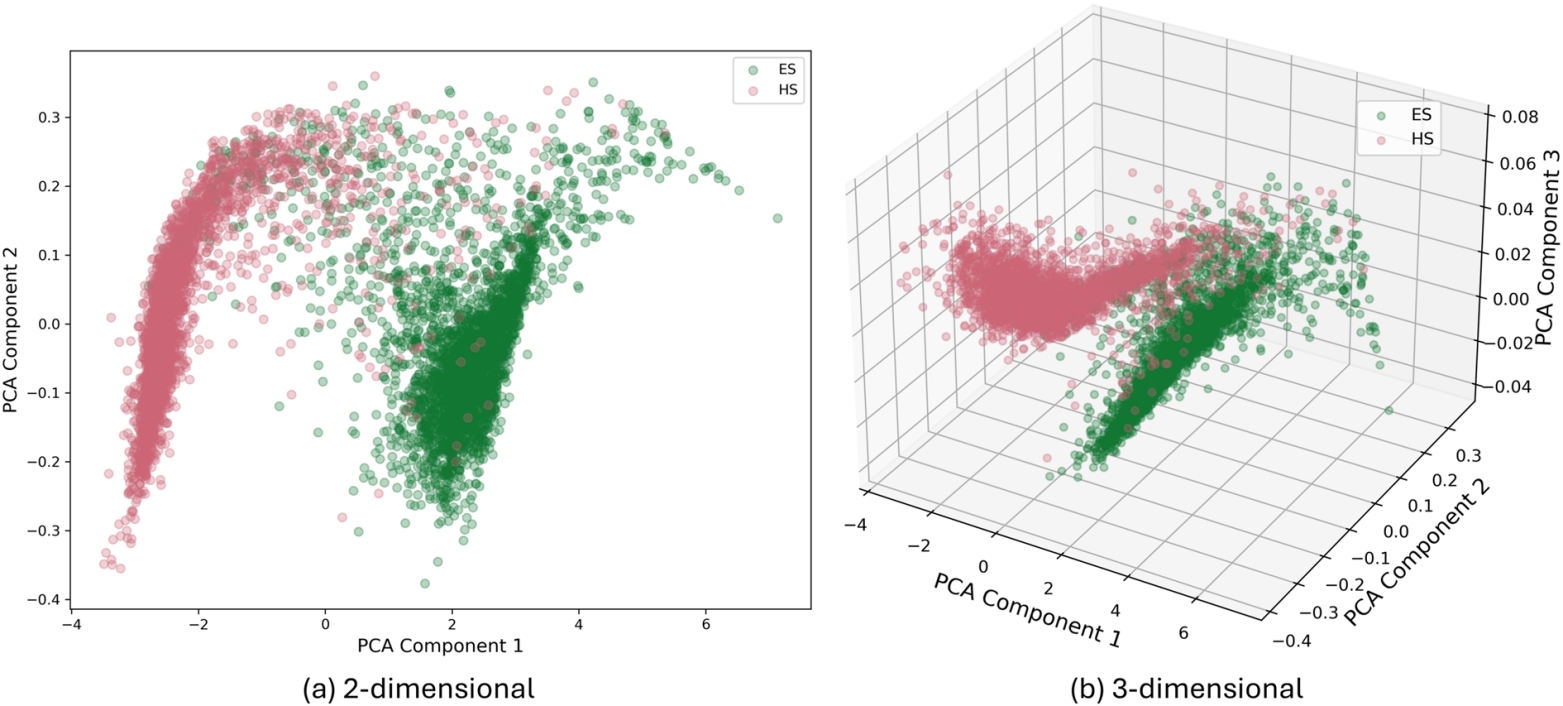
PCA of latent space for SECFP model. Molecules in latent space are colored according to its class - ES/HS

Figure 4 shows a linear projection (PCA) of the latent space in 2 and 3 dimensions (for the ES and HS dataset). PCA component 1 captures 99.7% of the variance in the latent space, while components 2 and 3 account for the remaining 0.3%. This indicates that the contrastive Hellinger loss identified a minimal discriminative subspace—a few key dimensions in the latent representation that lead to the separation of the ES/HS classes. The remaining dimensions contain similar values for both classes, becoming irrelevant to the classification. Hence, the model effectively identified the most distinguishing molecular features between the two classes.

The PCA analysis confirms the success of contrastive training and aligns with the observed separation of ES/HS molecule prices in the previous section.

The 2D and 3D PCA for the Morgan fingerprint can be found in the SI.

### Applicability of MolPrice

#### Comparison to SA tools

To showcase *MolPrice*’s use case as an SA scoring tool, we benchmarked and compared it to other state-of-the-art tools as shown in Table 3. *MolPrice* performs competitively for both fingerprint types on TS1/2. *MolPrice* based on the SECFP-hybrid fingerprint outperforms its Morgan-hybrid counterpart on the TS3 dataset by a significant margin (similar to Table 2). Although *MolPrice* outperforms most models on TS1-3 (for F-Score and ROC-AUC), it fails to outperform *DeepSA* and *BR-SAScore*. Despite this fact, there are some points that should be kept in mind while analyzing the results.

1. *DeepSA* and *BR-SAScore* are models developed exclusively for SA scoring. For *DeepSA* (and *GASA*), the sole objective is to maximize the binary classification performance. *BR-SAScore* introduces reaction insights within the “likeliness-based” *SAScore*, improving an already widely accepted SA scoring tool. *MolPrice* is not trained directly on a binary classification loss and does not build upon previous SA-tools. Instead, *MolPrice* learns to distinguish the molecules based on its latent space representation while ensuring a good predictive performance for molecular prices. These two objectives could be conflicting for some molecules as outlined previously.
2. Test sets TS2/3 are not labeled by humans as ES/HS, but rather by an *imperfect* CASP tool. Some compounds may have been mislabeled due to the failure of finding a synthesis route. The CASP tool is highly dependent on its hyperparameters (such as iteration budget, tree width) as well as the database it was trained and the building block dataset. Thus, running the HS dataset with different hyperparameters/building blocks will classify some compounds as ES, and vice versa for the ES dataset. This fact was observed for the *BR-SAScore* [14]. In particular, the authors of *BR-SAScore* relabeled molecules in TS1-3 using Retro* [44]—the same tool used by Ref. [10] for labeling TS2/3. For TS3, the authors found that 90 molecules previously labeled as ES are, in fact, HS [14]. Thus, running Retro* with different parameters evidently leads to a different classification. We re-computed the *BR-SAScore* for the dataset split used by GASA/DeepSA and found that the performance for TS3 dropped from 0.900 to 0.861 for ROC-AUC (Table 3).

**Table 3:** Performance comparison with other SA tools on three external test sets provided by Ref. [10]

Category	Method	ROC-AUC			F-Score			Speed
		TS1	TS2	TS3	TS1	TS2	TS3	(ms/mol)
Learning-based	DeepSA [16]	**1.000**	0.913	**0.896**	**0.995**	0.799	0.808	17.2
	GASA [10]	**1.000**	0.876	0.849	0.987	0.740	0.729	307
	RAScore [21]	0.982	0.865	0.790	0.914	0.625	0.656	123
	SYBA [11]	0.998	0.862	0.790	0.964	0.716	0.523	0.28*
	SCScore [23]	0.641	0.373	0.425	0.641	0.373	0.578	0.8
Likeness-based	SAScore [12]	0.999	0.919	0.772	0.989	0.737	0.333	0.39*
	BR-SAScore [14]	0.999	**0.961**	0.861	0.993	**0.884**	**0.811**	0.42*
*Ours* | **MolPrice**	Morgan-Hybrid	0.991	0.887	0.666	0.975	0.807	0.565	1.3
	SECFP-Hybrid	0.998	0.879	0.839	0.987	0.788	0.758	1.3

Scores and Speed for other SA tools were obtained from Refs. [14, 16], respectively

The best value is highlighted in bold. MolPrice’s best is underlined

*Algorithm not based on deep learning architecture

Looking at the inference time (speed) of the different molecules, *MolPrice* is slower than the likeness-based methods and *SYBA*. These three methods are not based on deep learning architectures and thus are naturally computationally efficient. Compared to other deep learning SA methods, such as *GASA*, *RAScore* and *DeepSA*, *MolPrice* is seen to have a considerable speed advantage, allowing it to screen ten to hundred times more molecules within the same time frame.

From Table 3, we can conclude that *MolPrice* is a competitive SA scoring tool. *MolPrice* could thus be used as a proxy for SA in molecular generative design routines and virtual screening. We highlight one of its use-cases in the following section.

#### Virtual screening case study

**Fig. 5:**
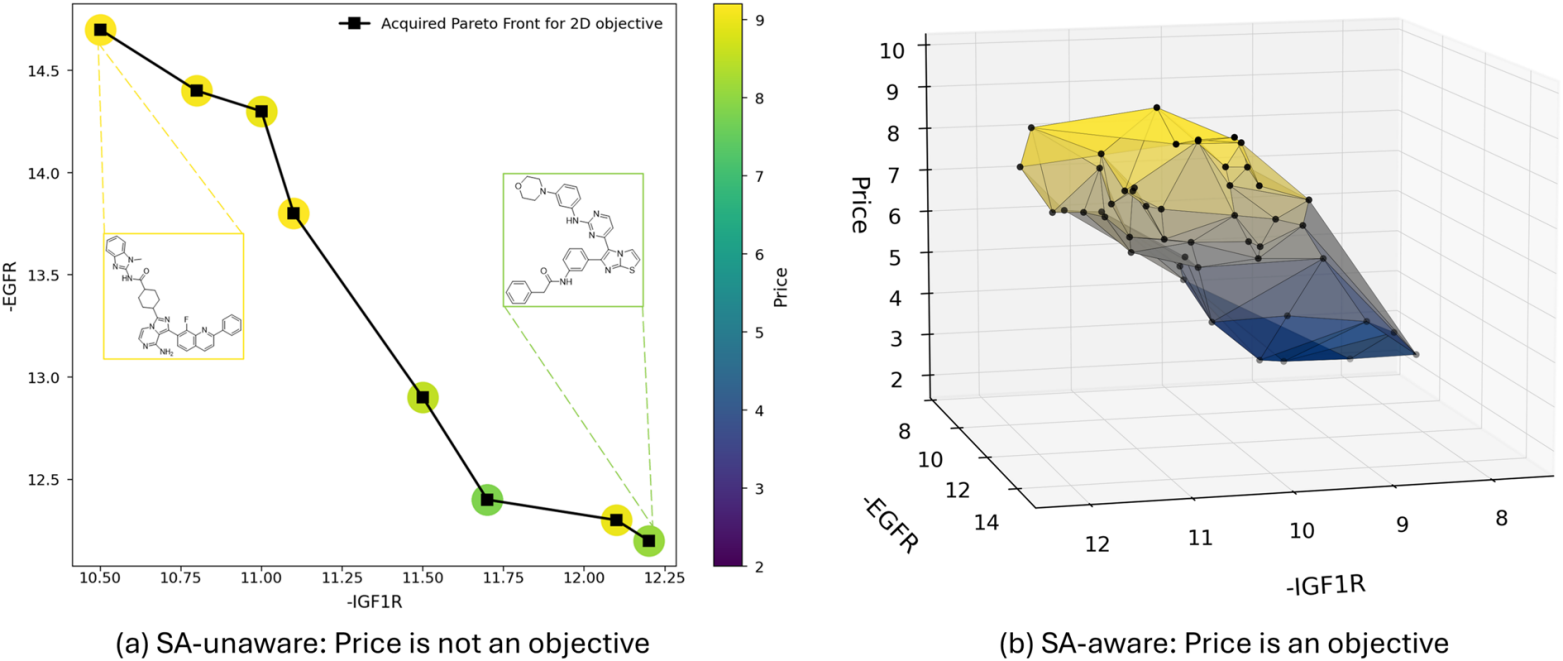
Pareto front of the virtual screening case study. Each point on the front represents a ligand selected from the dataset during the screening routine

The virtual screening routine was carried out twice, once for the SA-unaware case producing a 2D Pareto front (the objectives being ligand activity on 2 different proteins) and for the SA-aware case producing a 3D front (using *MolPrice* with the SECFP-hybrid fingerprint). Figure 5 displays both Pareto fronts. Each point on the front represents a ligand that was selected by *MolPAL* during the screening routine. A color bar is added to subplot Fig. 5a to indicate the price value of a molecule on the front. By visual inspection, one can see that all the molecules for the SA-unaware case are on the higher end of the price range (8–9), potentially missing out on cheaper alternatives with similar activities for the two proteins.

Table 4 presents the averaged statistical metrics for all ligands on the Pareto front. The purchasability metric is expressed as a percentage, representing the maximum Tanimoto similarity between each ligand and molecules in the Mcule database based on the Morgan fingerprint. A Tanimoto similarity exceeding 70% indicates that the ligand shares a similar chemical scaffold with existing compounds, while a similarity of 90% or higher suggests that the ligand likely exhibits comparable pharmacological activities to known molecules. Additionally, synthesis route analysis was performed for each ligand, yielding three key metrics: route existence (whether a viable synthetic pathway could be identified), route complexity (measured by the minimum number of convergent steps required for synthesis), and route reliability (which indicates the degree to which the predicted reactions are supported by established literature precedence).

**Table 4:** Averaged statistics calculated for all ligands on Pareto front for SA-aware and -unaware cases

Cases	Purchasability			Route statistics		
	>70%	>90%	Exact	Route existence ($\uparrow$)	Route complexity ($\downarrow$)	Route reliability ($\uparrow$)
SA-unaware	0.67	0.0	0.0	0.55	3.6	0.80
SA-aware	0.91	0.42	0.33	0.73	2.1	0.63

Purchasability % refers to the Tanimoto similarity of ligands to purchasable compounds

For both cases, *MolPAL* returned 18,000 ligands from the virtual library and constructed the optimal Pareto front based on the objectives. For the SA-unaware case, 67% of the ligands on the Pareto front demonstrate greater than 70% similarity with purchasable compounds, though none reach the desired 90% similarity threshold. In contrast, the SA-aware approach shows substantial improvement, with 91% of ligands having similar purchasable counterparts and 42% exceeding 90% similarity. Notably, 33% of ligands on the SA-aware Pareto front have exact matches in the Mcule database, indicating their immediate commercial availability. Hence, one can directly purchase and test a molecule in the lab, without having to undergo laborious synthesis. The route statistics further support this trend. In the SA-aware cases, ASKCOS successfully identified synthetic routes for 73% of molecules on the Pareto front, with an average route depth of 2.1. In contrast, the SA-unaware case achieved a lower success rate of 55% and a higher average depth of 3.6. A lower route depth is generally associated with a higher likelihood of successful synthesis, as each additional reaction step introduces uncertainty. Interestingly, the SA-unaware case yielded a higher average reliability score based on literature precedence, suggesting that its proposed reactions align more closely with documented precedents. However, ASKCOS does not provide estimates for reaction yield or selectivity, making it difficult to assess the practical feasibility of these routes. Routes with fewer steps remain more promising, as they inherently reduce the cumulative uncertainty associated with multiple reactions.

**Fig. 6:**
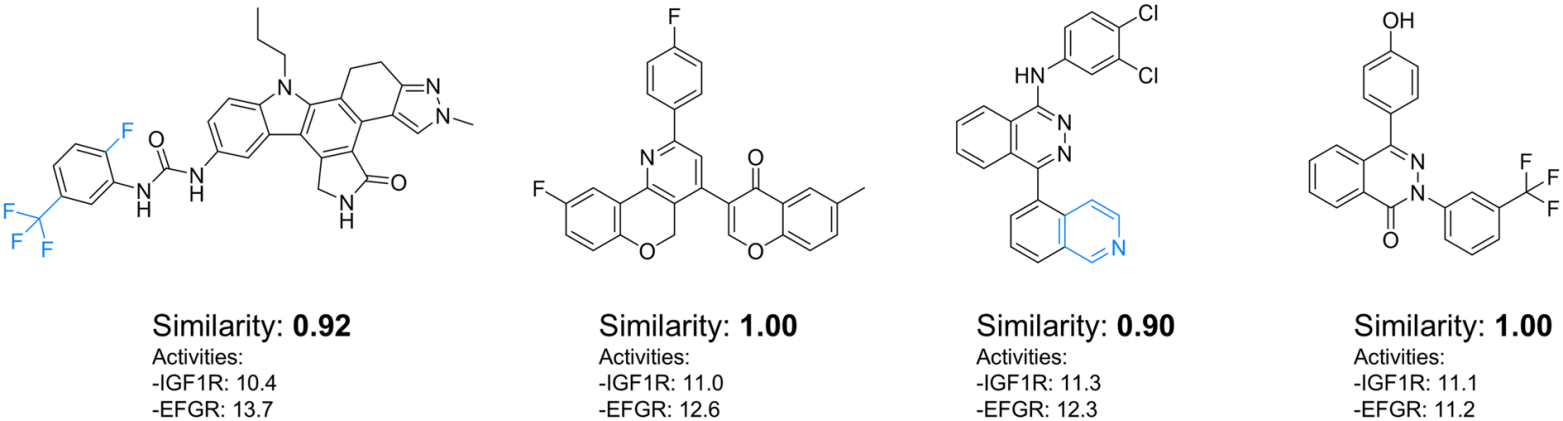
Exemplary molecules picked from the 3D Pareto Front for the SA-aware case study with a high (or exact) similarity to purchasable compounds and high activity for the two proteins. Moieties highlighted in blue are the not present in the purchasable compound

Figure 6 shows four exemplary molecules from the 3D Pareto front for the SA-aware case study. Two of these molecules match exactly with purchasable compounds from the Mcule database. The other two molecules have an extremely similar counterpart in the Mcule database and would be expected to have similar pharmacological activities. None of the molecules were selected in the SA-unaware case, as they are dominated points on the 2D Pareto front. As aforementioned, one could purchase these molecules and test them directly in the laboratory. In the SA-unaware case, one would have to spend time and energy to synthesize the molecules, slowing down the discovery.

As a final note, the DOCKSTRING dataset consists of ligands that obey the Lipinski’s rule of five, i.e., all ligands are drug-like. Truely hard-to-synthesize molecules are therefore rare within the DOCKSTRING benchmark. In the future, it would be of interest to extend the virtual screening case study to a dataset with a higher percentage of “undrug-like” molecules.

## Conclusion & outlook

This work introduces *MolPrice*, a fast and accurate deep learning model for molecular price prediction and evaluation of synthetic accessibility. Leveraging self-supervised contrastive learning, *MolPrice* effectively distinguishes between purchasable and hard-to-synthesize molecules based on their latent space representations. Our comprehensive experimentation with various molecular representation schemes reveals the importance of molecular substructures for accurate price prediction. When benchmarked against widely accepted synthetic accessibility tools, *MolPrice* demonstrates competitive performance, although not achieving state-of-the-art performance. The virtual screening case study further illustrates *MolPrice*’s effectiveness in prioritizing molecules that are readily synthesizable and/or accessible through chemical suppliers, thereby accelerating the drug discovery process. A key advantage of *MolPrice* lies in its fast inference time allowing the model to calculate prices for large libraries of molecules within a short timeframe. Compared to the literature price prediction model, *CoPriNet* [22], and deep learning based SA methods, *MolPrice* experiences a ten- to hundred-fold speed up, making it particularly valuable for market-aware, large-scale screening. Furthermore, its quick training time allows for regular model updates as new molecules are added to both purchasable compound databases and hard-to-synthesize datasets.

In future work, we aim to integrate *MolPrice* as a guide tool for generative molecular design routines [48] and as an objective function for synthesis route planning [49]. Of particular interest is whether incorporating molecular price estimation into the objectives of synthesis planners leads to faster convergence in computer-aided synthesis planning (CASP) tools. Because lower-priced molecules generally correlate with higher commercial availability, CASP algorithms that explicitly optimize for price may more efficiently identify purchasable building blocks, reducing the number of iterations to find a viable synthesis route.

Ultimately, by bridging the gap between molecular design and synthetic feasibility through price-based metrics, *MolPrice* offers a practical approach to accelerate the discovery-to-synthesis pipeline, potentially reducing both the time and resources required to bring new chemical entities from conceptualization to reality.

## Supplementary Information

Supplementary material 1.

## Data Availability

The code repository supporting the presented work can be found via GitHub: https://github.com/OptiMaL-PSE-Lab/MolPrice. Datasets and model checkpoints are provided via FigShare: https://doi.org/10.6084/m9.figshare.c.7729217.v1
