# Supplementary material for: MolPrice: assessing synthetic accessibility of molecules based on market value
Source: J Cheminform. 2025 Sep 29;17:150. doi: 10.1186/s13321-025-01076-3 (PMC12482503; doi:10.1186/s13321-025-01076-3)
Supplement: Supplementary file 1 — Supplementary material 1. [file 13321_2025_1076_MOESM1_ESM.pdf]

Supplementary Information (SI)

S1 Dataset Statistics

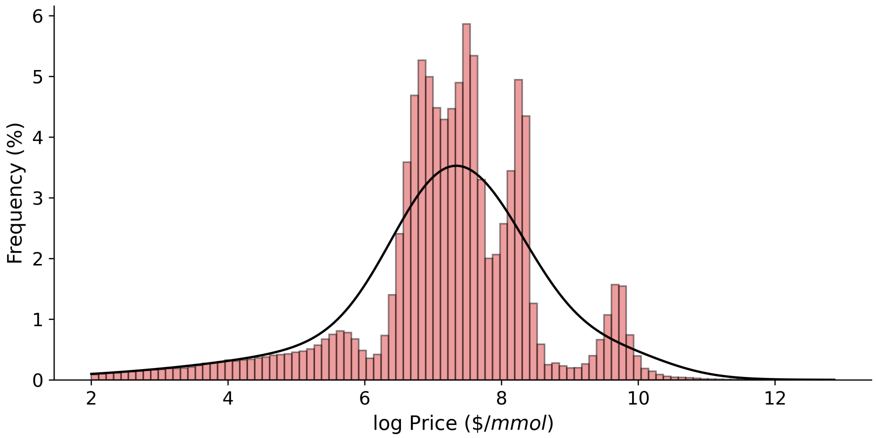

Figure S1: Distribution of Molecular Prices in Molport Dataset after removing all prices that are $< 2\$/mmol$

S2 Additional Methodological Details

S2.1 Complexity Indicators from RDKit

Below is a list and explanation of the 10 complexity indicators used for the Hybrid fingerprints:

1. **FractionCSP3** returns the fraction of atoms that are sp^3 hybridized

2. **NumAtomStereoCenters** returns the total number of atomic stereocentres

3. **NumRotableBond** returns the total number of rotable bonds

4. **NumHeterocycles** returns the total number of heterocycles

5. **NumSpiroAtoms** returns the total number of spiro atoms (atoms shared between rings that share exactly one atom)

6. **NumBridgeheadAtoms** returns the number of bridgehead atoms (atoms shared between rings that share at least two bonds)

7. **NumMacrocyles** returns the number of macrocycles (rings with more than 6 atoms)

8. **NumMultiRingAtoms** returns the number of atoms that belong to multiple rings

9. **SPS** calculates the Spacial Score, as introduced in Krzyzanowski et al.[1]

10. **TPSA** estimates and returns the polar surface area

S2.2 Model Parameters

Table S1: Hyperparameters for different model architectures investigated for the price prediction task. Parameters for *CoPriNet* can be obtained from Sanchez-Garcia et al.[2]

Model Type	**No. Layers**	**No. Heads**	**Hidden Dim.**	**Dropout**	**Input Size**	**LR**	**Batch S.**
MLP	4	N/A	[512, 256, 128]	0.2	4096	2e-4	256
Transformer	3	4	600	0.12	500	1e-4	32
RoBERTa	3	12	384	0.109	600	1e-5	64
LSTM (EFG)	3	N/A	200	0.1	7000	1.5e-4	256

S2.3 ASKCOS v2 search parameters

As retrosynthesis software is highly dependent on the underlying search model, we list our configuration below that was used to generate route statistics for the virtual screening case study:

Single-step model: Template - relevance (Reaxys)

Tree Builder: Monte Carlo Tree Search (MCTS)

Max. Depth: 5

Max. Branching Factor: 25

Max Search Time: 60 sec

S3 Additional Results

S3.1 Latent Space Distribution

As shown in Figure S2, the latent dimensions undergo slight changes during contrastive learning. However, because the readout function is frozen, a decrease in contribution from certain dimensions is compensated by increases in others. This redistribution preserves the overall validity of the learned mapping from latent space to price.

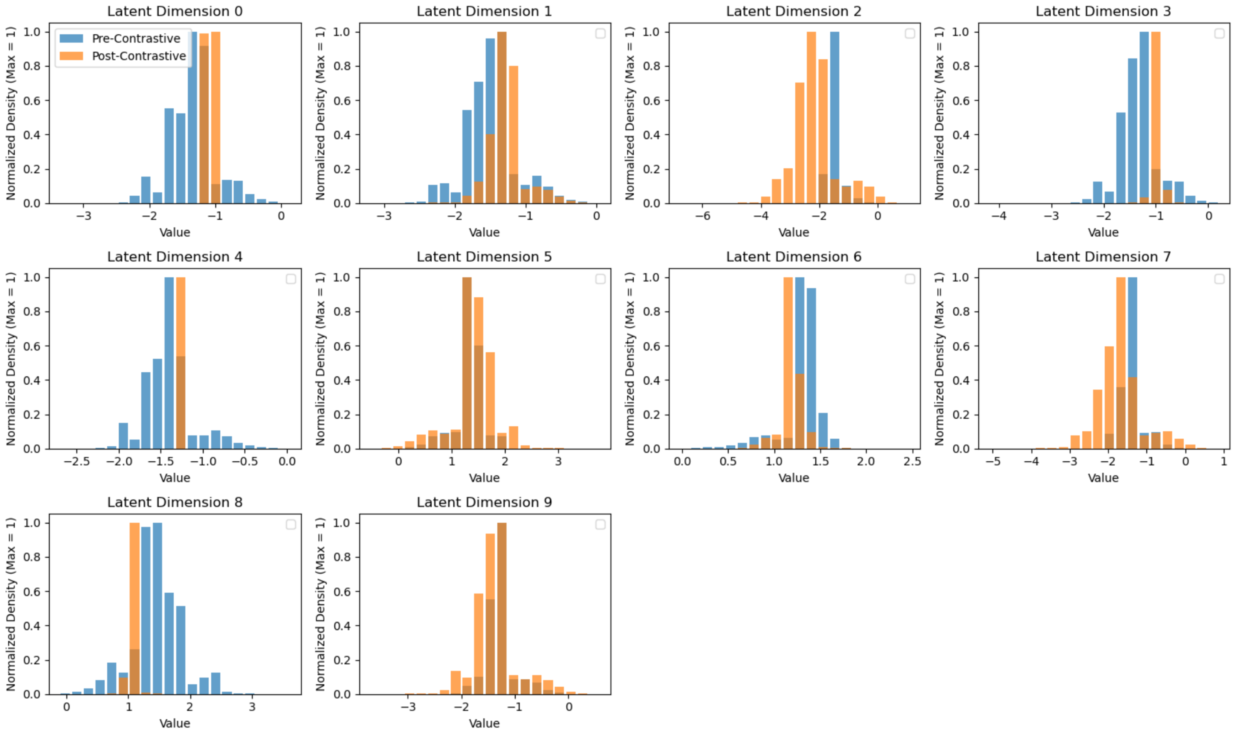

Figure S2: Distribution of latent representation pre- and post-contrastive learning for 50,000 randomly selected ES molecules from the purchasable test dataset

S3.2 MolPrice with COCONUT database as HS training database

Instead of the HS databases created with Nonpher by Yu et al.[3], we utilize the natural product (NP) database by COCONUT[4]. In doing so, *MolPrice-NP* learns to distinguish between ES and NP molecules. In the future, it may be worth investigating if the output by *MolPrice-NP* correlates well with other NP likelihood scores.

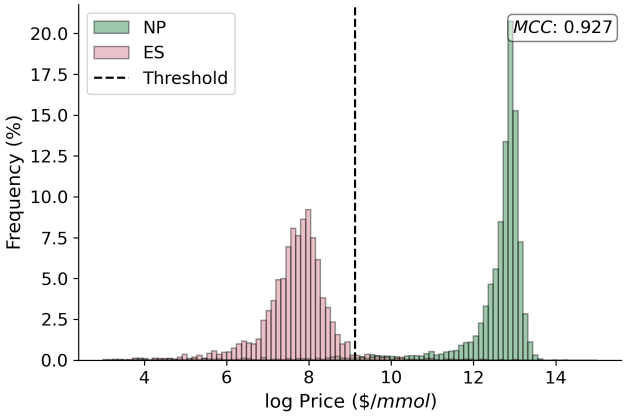

Figure S3: MolPrice is capable of telling apart ES and NP molecules after self-supervised contrastive learning taking COCONUT's NP database[4] as the unlabeled, complex molecule dataset

S3.3 Hyperparameter Tuning - Parameter Analysis

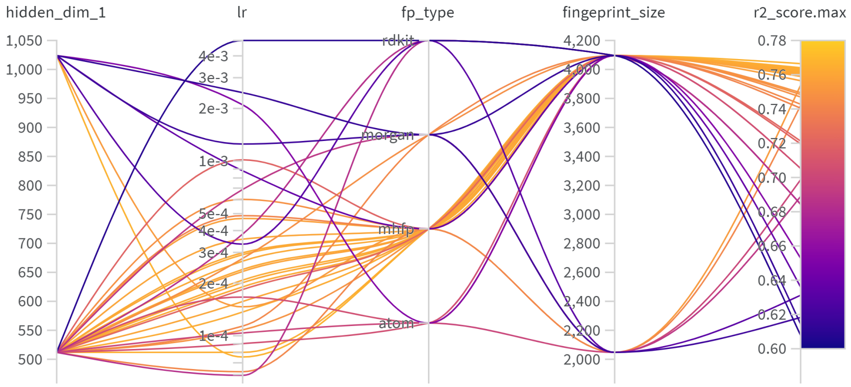

Figure S4: Overview of all individual runs for hyperparameter tuning. The objective is to find the best set of parameters that optimize the coefficient of determination (R^2)

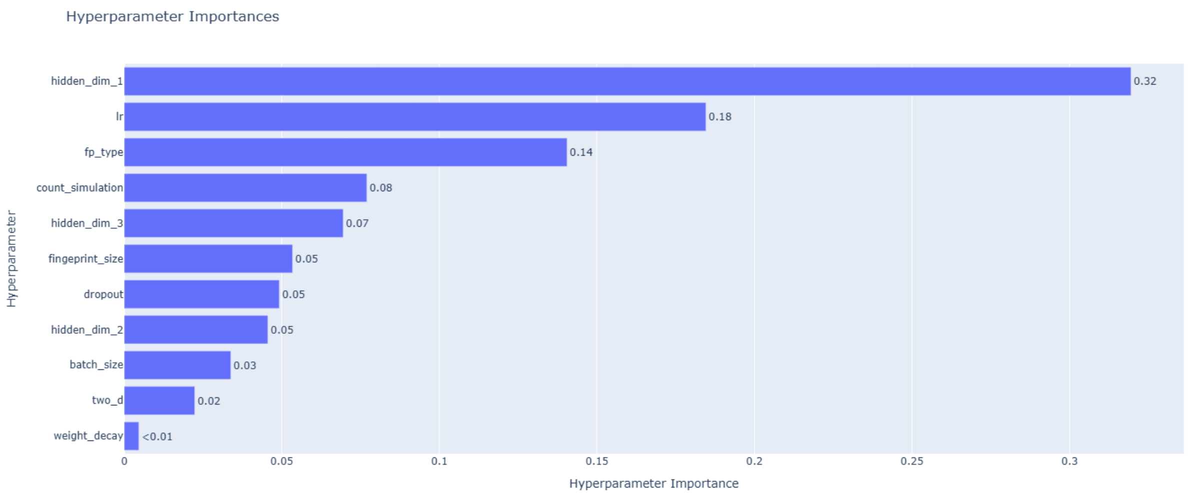

Figure S5: Hyperparameter importance according to Optuna's Tree-structure Parzan Estimator

S3.4 PCA projection for Morgan-Hybrid fingerprint

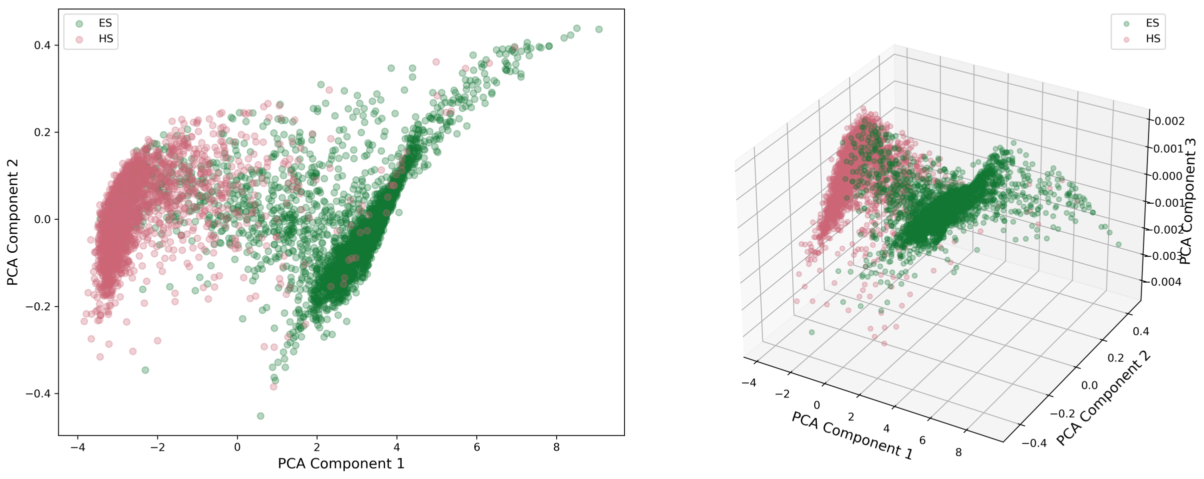

(a) 2-dimensional

(b) 3-dimensional

Figure S6: PCA of latent space for Morgan model. Molecules in latent space are colored according to its class - ES/HS.

References

[1] Adrian Krzyzanowski, Axel Pahl, Michael Grigalunas, and Herbert Waldmann. Spacial score - a comprehensive topological indicator for small-molecule complexity. *Journal of Medicinal Chemistry*, 66(18):12739–12750, 2023.

[2] Ruben Sanchez-Garcia, Dávid Havasi, Gergely Takács, Matthew C. Robinson, Alpha Lee, Frank von Delft, and Charlotte M. Deane. CoPriNet: Graph neural networks provide accurate and rapid compound price prediction for molecule prioritisation. *Digital Discovery*, 2(1):103–111, 2023. ISSN 2635-098X. doi: 10.1039/D2DD00071G.

[3] Jiahui Yu, Jike Wang, Hong Zhao, Junbo Gao, Yu Kang, Dongsheng Cao, Zhe Wang, and Tingjun Hou. Organic compound synthetic accessibility prediction based on the graph attention mechanism. *Journal of Chemical Information and Modeling*, 62(12):2973–2986, 2022. ISSN 1549-9596. doi: 10.1021/acs.jcim.2c00038.

[4] Venkata Chandrasekhar Nainala, Kohulan Rajan, Sri Ram Sagar Kanakam, Nisha Sharma, Viktor Weißenborn, Jonas Schaub, and Christoph Steinbeck. Coconut 2.0: A comprehensive overhaul and curation of the collection of open natural products database, 2024. preprint.
